# Supplementary material for: Modeling glioblastoma heterogeneity as a dynamic network of cell states
Source: Mol Syst Biol. 2021 Sep 16;17(9):e10105. doi: 10.15252/msb.202010105 (PMC8444284; doi:10.15252/msb.202010105)
Supplement: Supplementary file 6 — Source Data for Figure 5 [file MSB-17-e10105-s004.zip › Figure5A_sourcedata/GSEA_3071/hallmarks_stateVI.GseaPreranked.1623742557031/HALLMARK_MYC_TARGETS_V2.html]

Details for gene set HALLMARK\_MYC\_TARGETS\_V2[GSEA]

|  || Dataset | state63071 |
| Phenotype | NoPhenotypeAvailable |
| Upregulated in class | na\_pos |
| GeneSet | HALLMARK\_MYC\_TARGETS\_V2 |
| Enrichment Score (ES) | 0.4284382 |
| Normalized Enrichment Score (NES) | 2.455661 |
| Nominal p-value | 0.0 |
| FDR q-value | 0.0 |
| FWER p-Value | 0.0 |
Table: GSEA Results Summary

  

Fig 1: Enrichment plot: HALLMARK\_MYC\_TARGETS\_V2      
 Profile of the Running ES Score & Positions of GeneSet Members on the Rank Ordered List

  

| PROBE | GENE SYMBOL | GENE\_TITLE | RANK IN GENE LIST | RANK METRIC SCORE | RUNNING ES | CORE ENRICHMENT || 1 | MYC |  |  | 69 | 0.815 | 0.0306 | Yes |
| 2 | NPM1 |  |  | 114 | 0.717 | 0.0622 | Yes |
| 3 | WDR43 |  |  | 162 | 0.644 | 0.0885 | Yes |
| 4 | IMP4 |  |  | 208 | 0.597 | 0.1124 | Yes |
| 5 | MRTO4 |  |  | 229 | 0.582 | 0.1425 | Yes |
| 6 | SRM |  |  | 292 | 0.544 | 0.1585 | Yes |
| 7 | RABEPK |  |  | 295 | 0.544 | 0.1911 | Yes |
| 8 | NIP7 |  |  | 308 | 0.537 | 0.2206 | Yes |
| 9 | PES1 |  |  | 316 | 0.530 | 0.2510 | Yes |
| 10 | NOP16 |  |  | 392 | 0.492 | 0.2602 | Yes |
| 11 | WDR74 |  |  | 504 | 0.454 | 0.2571 | Yes |
| 12 | PPRC1 |  |  | 682 | 0.404 | 0.2326 | Yes |
| 13 | IPO4 |  |  | 716 | 0.393 | 0.2474 | Yes |
| 14 | BYSL |  |  | 726 | 0.391 | 0.2688 | Yes |
| 15 | NOP2 |  |  | 821 | 0.373 | 0.2655 | Yes |
| 16 | RRP9 |  |  | 872 | 0.365 | 0.2739 | Yes |
| 17 | TBRG4 |  |  | 884 | 0.363 | 0.2930 | Yes |
| 18 | NDUFAF4 |  |  | 908 | 0.360 | 0.3085 | Yes |
| 19 | PHB |  |  | 918 | 0.357 | 0.3279 | Yes |
| 20 | PUS1 |  |  | 933 | 0.355 | 0.3457 | Yes |
| 21 | AIMP2 |  |  | 973 | 0.349 | 0.3562 | Yes |
| 22 | MYBBP1A |  |  | 980 | 0.349 | 0.3758 | Yes |
| 23 | PA2G4 |  |  | 986 | 0.348 | 0.3957 | Yes |
| 24 | UTP20 |  |  | 1014 | 0.343 | 0.4091 | Yes |
| 25 | TFB2M |  |  | 1072 | 0.334 | 0.4137 | Yes |
| 26 | EXOSC5 |  |  | 1192 | 0.317 | 0.4000 | Yes |
| 27 | RRP12 |  |  | 1217 | 0.313 | 0.4124 | Yes |
| 28 | SORD |  |  | 1239 | 0.311 | 0.4256 | Yes |
| 29 | PPAN |  |  | 1304 | 0.303 | 0.4263 | Yes |
| 30 | NOC4L |  |  | 1380 | 0.294 | 0.4234 | Yes |
| 31 | FARSA |  |  | 1452 | 0.285 | 0.4211 | Yes |
| 32 | NOP56 |  |  | 1531 | 0.276 | 0.4163 | Yes |
| 33 | HSPD1 |  |  | 1556 | 0.274 | 0.4263 | Yes |
| 34 | PLK1 |  |  | 1665 | 0.263 | 0.4123 | Yes |
| 35 | NOLC1 |  |  | 1699 | 0.260 | 0.4191 | Yes |
| 36 | PRMT3 |  |  | 1723 | 0.258 | 0.4284 | Yes |
| 37 | CBX3 |  |  | 1983 | -0.275 | 0.3733 | No |
| 38 | UNG |  |  | 2284 | -0.330 | 0.3101 | No |
| 39 | MCM5 |  |  | 2424 | -0.359 | 0.2934 | No |
| 40 | MCM4 |  |  | 3254 | -0.715 | 0.1067 | No |
Table: GSEA details [plain text format]

  

Fig 2: HALLMARK\_MYC\_TARGETS\_V2: Random ES distribution      
 Gene set null distribution of ES for **HALLMARK\_MYC\_TARGETS\_V2**
